# Evaluating the Effect of TeamSTEPPS on Teamwork Perceptions and Patient Safety Culture among Newly Graduated Nurses

**DOI:** 10.1186/s12912-024-01850-y

**Published:** 2024-03-13

**Authors:** Asmaa Elwan Hassan, Faten Ahmed Mohammed, Abeer Mohamed Zakaria, Ibrahim Abdullatif Ibrahim

**Affiliations:** Department of nursing administration, Faculty of nursing, Mansoura University, Mansoura, Egypt

**Keywords:** Patient safety culture, Nurses, TeamSTEPPS, Teamwork

## Abstract

**Background:**

Quality healthcare delivery is contingent upon effective teamwork and a patient safety-focused culture. TeamSTEPPS offers an evidence-based framework that enhances these competencies. However, the impact of TeamSTEPPS on newly graduated nurses, who undergo a significant transitional phase, has yet to be comprehensively explored. Consequently, the objective of this study was to assess the influence of TeamSTEPPS on perceptions of teamwork and patient safety culture among newly graduated nurses.

**Methods:**

This study employed a quasi-experimental pretest-posttest design with a single group, utilizing a convenience sample of 132 newly recruited nurses from a university hospital. The participants completed the hospital survey on patient safety culture and the TeamSTEPPS teamwork perceptions questionnaire at three different time points.

**Results:**

The impact of the TeamSTEPPS training program was found to be significant, as indicated by the substantial improvement in the mean scores of nurses’ perceptions regarding teamwork and the culture of patient safety across multiple assessments (*p* < 0.001). The effect size (η2p ≥ 0.14) suggests a large effect, further emphasizing the meaningful impact of the program on the measured outcomes.

**Conclusions:**

The study underscores the effectiveness of TeamSTEPPS as a valuable framework for facilitating the seamless transition of newly graduated nurses into the healthcare field. Integrating TeamSTEPPS into nursing training programs can significantly enhance nurses’ perceptions of teamwork and the culture of patient safety. Therefore, it is crucial for nurse managers to implement TeamSTEPPS systematically, aiming to improve teamwork perception and cultivate a patient safety culture among nurses. Furthermore, they should establish mechanisms to ensure the consistent application of these skills over time.

## Introduction

Effective communication and teamwork﻿ serve as vital ingredients while offering quality care in a healthcare setting [1]. Collaborative clinical teams that employ clear and direct interpersonal exchanges can effectively enhance health outcomes [2, 3]. However, with the increasing complexity of care provision, there is an urgent need for structured interventions that can strengthen team cohesion and foster a culture focused on safety [4]. One evidence-based model that effectively addresses this need is Team Strategies and Tools to Enhance Performance and Patient Safety (TeamSTEPPS). Developed through a collaborative effort between the Agency for Healthcare Research and Quality (AHRQ) and the Department of Defense, TeamSTEPPS combines the expertise from both the healthcare and military sectors to create a comprehensive framework for improving teamwork and enhancing patient safety [5]. TeamSTEPPS can be defined as an evidence-based approach that offers a comprehensive set of tools and strategies. These resources are designed to facilitate the transition, enhance effective teamwork and communication, and foster a culture of safety and quality care [6]. TeamSTEPPS utilizes a systematic framework centered around four core competencies: communication, leadership, situation monitoring (actively assessing situational elements such as patient status and environment), and mutual support (anticipating and addressing the needs of team members) [7, 8]. Furthermore, TeamSTEPPS provides a comprehensive range of fifteen tools and strategies specifically designed for implementation in clinical practice. These tools and strategies aim to enhance collaboration, communication, and ultimately improve patient safety outcomes among healthcare professionals [9].

Several studies have been conducted to investigate the implementation and outcomes of TeamSTEPPS interventions in diverse healthcare settings. One notable study by Matzke et al. focused on assessing the effects of TeamSTEPPS on teamwork perceptions and communication among clinical staff members in an academic Level I Emergency and Trauma Center. The findings of this study revealed significant improvements in teamwork attitudes, communication, and collaboration, thereby highlighting the positive influence of the TeamSTEPPS program on team dynamics [3]. Furthermore, research studies have highlighted the importance of TeamSTEPPS in reducing medical errors and adverse events. For instance, an integrative review conducted by Parker et al. examined the impact of TeamSTEPPS on patient safety, clinical errors associated with communication, and patient satisfaction. The findings of this review demonstrated that the implementation of TeamSTEPPS can lead to improvements in communication, reductions in clinical error rates, and enhanced patient satisfaction [2].

For newly graduated nurses embarking on their journey in dynamic healthcare environments, the integration of effective group approaches holds significant importance. The transition from education to practice requires not only clinical competence but also the capacity to navigate interprofessional teams and communicate effectively [10]. Teamwork can be defined as a cohesive combination of interconnected knowledge, skills, and attitudes that all team members must possess in order to operate efficiently as a collective unit [11]. The foundational components of the teamwork concept encompass team leadership, backup behavior, adaptability, team orientation, and reciprocal situation monitoring [11]. The perception of teamwork in the nursing environment has a profound impact on the quality of care provided to patients, the satisfaction of employees in their work, and the overall efficacy of healthcare services [12–14].

TeamSTEPPS has been shown to significantly improve nursing perceptions of teamwork within healthcare organizations [3, 15]. Several studies have underscored the positive impact of TeamSTEPPS interventions on nurses' perceptions and behavior within healthcare teams. Participation in TeamSTEPPS interventions has been found to enhance perceptions of teamwork, improve communication, collaboration, mutual respect, and shared accountability among nurses [3, 15–17]. This approach cultivates a favorable climate for nurse teamwork, rendering it an effective intervention for enhancing healthcare quality and patient outcomes. Integrating TeamSTEPPS principles into nursing practice has also been associated with reduced medical errors, improved patient safety culture, and enhanced job satisfaction [2, 18–20].

To our knowledge, exploring the impact of TeamSTEPPS on the newly graduated nurses’ perceptions towards teamwork and patient safety culture is an area that has not yet been thoroughly investigated especially among novice nurses in Egypt. Newly qualified nurses experience transformation from college life into a practice in a complicated healthcare system that provides distinct challenges for such adjustment [21]. This research has the potential to provide valuable insights into the advantages of implementing the TeamSTEPPS approach during the transition from education to practice. Additionally, it can contribute to the enhancement of healthcare delivery and the improvement of patient outcomes. Therefore, the aim of the current study is to contribute to the existing literature by evaluating the impact of TeamSTEPPS on the perceptions of recently graduated nurses regarding teamwork and the culture of patient safety in healthcare environments.

## Research hypotheses

H1: The implementation of TeamSTEPPS training program will significantly enhance teamwork perceptions among newly graduated nurses.

H2: The implementation of TeamSTEPPS training program will result in improvements in patient safety culture among newly graduated nurses.

### Theoretical framework

Albert Bandura's social cognitive theory offers a valuable framework for comprehending the processes involved in learning and behavior. According to Bandura, individuals acquire knowledge, skills, and attitudes by observing others and the outcomes they encounter. This theory highlights the significance of cognitive processes, such as attention, memory, and motivation, in mediating learning and behavior [22]. In the healthcare context, Bandura's social cognitive theory offers valuable insights into how TeamSTEPPS interventions can impact the perceptions and behaviors of newly graduated nurses. By enhancing self-efficacy beliefs in teamwork and communication, TeamSTEPPS interventions have the potential to empower nurses to engage in effective collaboration, problem-solving, and patient-centered care.

## Methods

### Study design

The study employed a quasi-experimental pretest–posttest design with a single-group [23]. The study adhered to the guidelines provided in the TREND Statement checklist [24]. The study protocol has been officially registered on ClinicalTrials.gov (Identifier code NCT06117800; 03/11/2023).

### Participants and setting

This study employed a convenience sample comprising newly registered nurses who had graduated within the past two years and were employed full-time in direct patient care roles. Nursing interns and nursing leaders were excluded from the study. Out of the initial 221 nurses invited to participate, 51 were excluded due to not meeting the specified criteria, 23 declined to participate, and 15 were used for piloting purposes. Ultimately, a total of 132 nurses were included in the study.

To recruit newly graduated nurses, a multifaceted recruitment strategy was implemented. Flyers describing the study's objectives and procedures were prominently displayed in the units where the target participants were stationed. Additionally, to maximize accessibility and convenience, flyers were strategically placed in the electronic signature areas of the hospital, including admission and discharge locations. Invitations were also shared in WhatsApp groups with the assistance of nurse managers.

The study was conducted at Mansoura University Hospital, which is a tertiary care teaching hospital affiliated with Mansoura University. This hospital, located in Mansoura City, holds the important role of being the primary teaching hospital for medical education programs.

### Outcomes and instruments

#### Team STEPPS Teamwork Perceptions Questionnaire (T-TPQ)

This questionnaire utilized in this study was originally developed and validated by Battles and King in 2010 [25]. Its primary objective was to assess the perceptions of newly graduated nurses regarding group-level team skills and behavior. The questionnaire consisted of 35 questions, which were categorized into five domains: team structure, communication, leadership, situational monitoring, and mutual support. Each domain comprised seven items. The participants provided their responses using a 5-point Likert scale, ranging from 1 (strongly disagree) to 5 (strongly agree).

#### Hospital Survey on Patient Safety Culture (HSPSC)

The HSPSC, developed by the AHRQ in 2004 [26], was utilized to assess the patient safety culture from the perspectives of recently graduated nurses. The survey consisted of 42 items distributed across 12 domains. These domains covered various aspects such as teamwork within units (4 items), supervisor/manager expectations & actions promoting patient safety (4 items), organizational learning (3 items), management support for patient safety (3 items), overall perceptions of patient safety (4 items), communication and feedback about errors (3 items), communication openness (4 items), frequency of vents reported (3 items), teamwork a cross units (3 items), staffing (4 items), handoffs & transitions (4 items), and non-punitive response to errors (3 items). To calculate the mean scores for the overall HSPSC, the negative items were subjected to reverse coding. The nurses' responses were measured using a 5-point Likert scale, ranging from 1 (strongly disagree or never) to 5 (strongly agree or always).

#### Sociodemographic questionnaire

This questionnaire included questions related to participants’ gender, marital status, education, age, and experience.

### Validity and reliability of the scales

The utilized measures underwent a robust translation process from English to Arabic following Beaton guidelines to ensure linguistic equivalence and cultural suitability. The process involved several essential steps to ensure the accuracy and appropriateness of the translated measures for the Arabic-speaking population. These steps encompassed forward translation, expert panel review, back-translation, pre-testing, and cognitive interviewing. Each step was thoroughly executed to maintain the highest level of precision and relevance in the translated questionnaires [27]. The content validity of the translated scales was confirmed by a panel of experts. The feedback obtained from the validation procedure was utilized to enhance the phrasing of the items, aiming to achieve optimal comprehension, prior to the finalization of the Arabic versions. Subsequently, the instruments that had been translated were subjected to a pilot testing phase involving 15 newly graduated nurses. The purpose of this phase was to identify any items that needed rephrasing in order to enhance comprehension within the specific sociocultural context being targeted. The Cronbach's α coefficient of the T-TPQ subscales was ranged between 0.88 to 0.95 [25]. In this study, the Cronbach's α coefficient of the T-TPQ was determined to be 0.92, while the Cronbach's α coefficient of the subscales ranged between 0.86 and 0.94 (Table 3). The Cronbach's α coefficient of the HSPSC subscales ranged between 0.67 and 0.89 [26]. In this study, The HSPSC had a Cronbach's α coefficient of 0.93 with the Cronbach's α coefficient of the subscales ranging from 0.74 to 0.92 (Table 4).

### Data collection

This study utilized paper-based questionnaires to measure newly graduated nurses’ perceptions of patient safety culture, group-level team skills, and behavior at three time points: before implementation of the TeamSTEPPS training program (T0), immediately after completion of the TeamSTEPPS training program (T1), and 2 months after implementation (T3). The data was collected at the beginning of April 2023 and finished at the end of August 2023. In the first session of the TeamSTEPPS training program, the nurses completed the T-TPQ and HSPSC to establish baseline perceptions of patient safety culture and nurses’ perceptions of group-level team skills and behavior. In addition to the T-TPQ and HSPSC, a demographic questionnaire was also administered to collect relevant information about the participating nurses. After that, immediately after the training program, the participants were administered the T-TPQ and HSPSC again to determine the changes in nurses’ perceptions of patient safety culture, group-level team skills, and behavior. Two months later, participants were given the T-TPQ and HSPSC a third time to assess their sustained effects.

### The intervention

The TeamSTEPPS training program intervention was delivered over seven weeks to newly graduated nurses at Mansoura University Hospital. The established framework of the AHRQ TeamSTEPPS program initiative served as the foundation for the content of the intervention curriculum [7]. The training programs included different teaching strategies, didactic lectures, discussions, and hands-on simulation exercises for each module. The first module, held in the initial week, introduced participants to fundamental TeamSTEPPS concepts, including patient safety culture, the TeamSTEPPS framework, and medical errors. Module 2, covering team structure, followed a similar format and schedule, teaching participants about healthcare team structures, clinical team responsibilities, and the multi-team system for resident care. Interactive "lifeboat exercises" were used to reinforce these concepts, promoting hands-on learning and interaction among participants. The lifeboat exercises were conducted as simulation activities aiming to evaluate participants' decision-making skills and teamwork abilities within a challenging scenario. During the lifeboat exercises, participants were organized into small groups and assigned distinct roles, such as leader, navigator, or resource manager. They were presented with diverse scenarios and engaged in collaborative discussions to determine the optimal course of action. Subsequent modules, spanning communication, leading teams, situation monitoring, and mutual support, were conducted in the following weeks. Each module featured hands-on exercises and activities tailored to its theme, such as SBAR, callouts, check-backs, handoffs, briefs, huddles, and debriefs, among others (Table 1). The 132 nurse participants were divided into three groups of 44 based on their work schedules. The primary investigator, who has an MSN in nursing administration, delivered training in a sizable conference room at Mansoura University Hospital. Each 4-h module included a 1-h didactic lecture, 15-min breaks, a topic-specific discussion, and practical exercises. Post-training certification was awarded to incentivize completion. Periodic email and WhatsApp reminders were aimed at sustaining commitment throughout the 7-week training period.

**Table 1:**
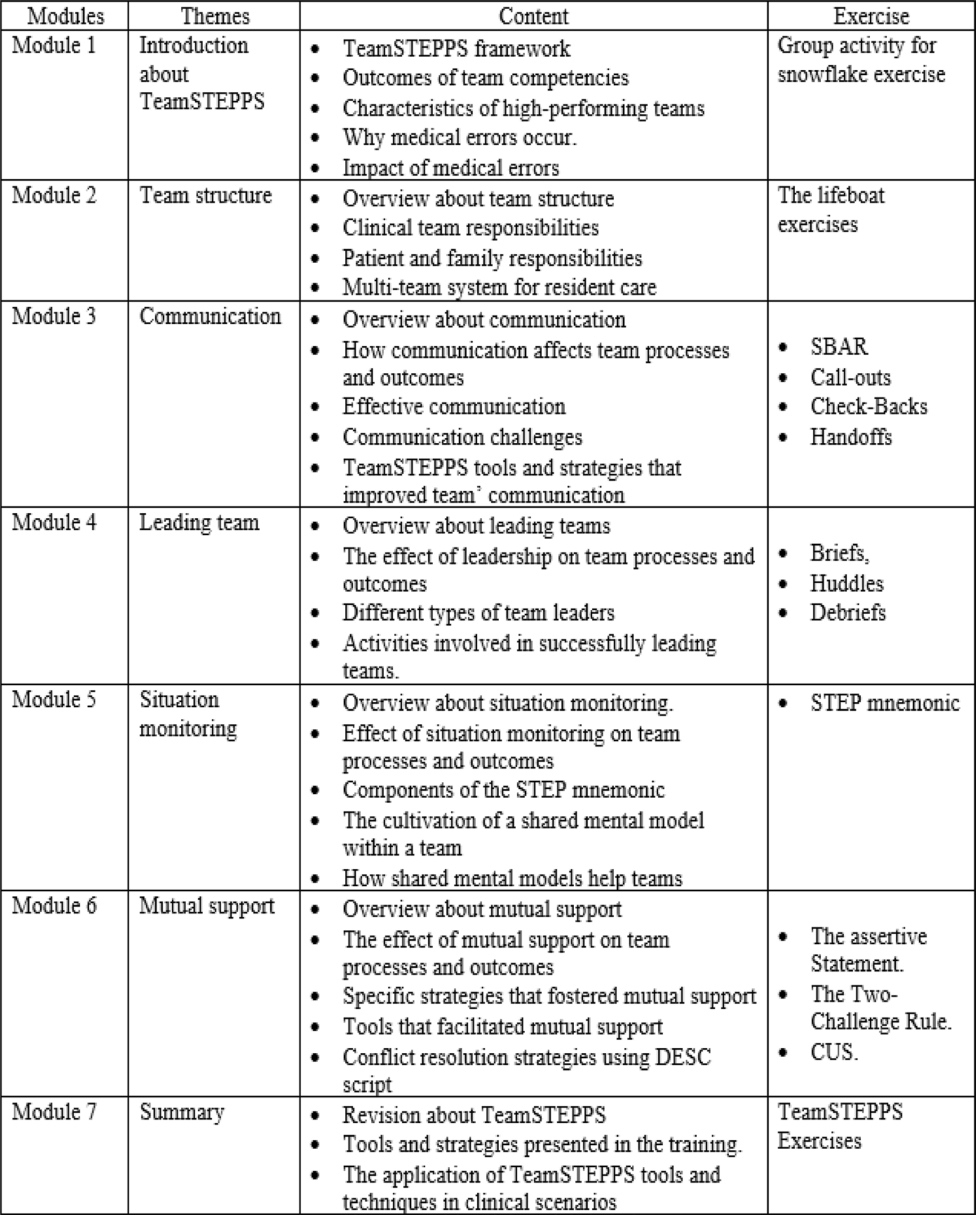
Structure of modules and content of TeamSTEPPS program

### Ethical considerations

This study adhered to the standards outlined in the Declaration of Helsinki [28]. Newly graduating nurses were informed about the study's nature and objectives, and their participation was contingent on informed consent. They were informed that their participation was voluntary, and they could withdraw at any time. Confidentiality of data and sample privacy were guaranteed to all participants.

### Statistical analysis

The collected data were organized, tabulated, and subjected to statistical analysis using SPSS.

software V23. The normality assumptions were confirmed before conducting statistical hypothesis testing. Absolute skewness and kurtosis values were computed for each variable at three timepoints following conventions in the literature that skewness less than 2 and kurtosis less than 4 demonstrate normality [29]. The central limit theorem asserts that when the sample size consists of 100 or more observations, deviations from normality are not a significant concern [30, 31]. Consequently, the categorical variables were depicted in terms of frequency and percentage. Continuous variables were expressed as the mean and standard deviation. A repeated measures ANOVA was performed to examine differences among several timepoints (T0, T1, and T2). The post-hoc paired t-test was employed to identify the particular locations within the dataset where significant changes occurred between timepoints. The effect size for the intervention was determined by calculating the partial eta-squared (η^2^p) for statistically significant differences. These differences were then categorized as small (0.01), medium (0.06), or large effect (≥ 0.14) [32]. The statistical analysis included two-sided tests, with a predetermined significance level of less than 0.05.

## Results

The study included a total of 132 newly graduated nurses. Out of these nurses, 72.0 percent were female, 73.5 percent were married, and 65.9 percent held a bachelor’s degree. The mean age of the participants was estimated to be 21.92 years (SD = 0.89). Furthermore, the average duration of professional experience was found to be 10.98 months (SD = 6.41). Throughout the TeamSTEPPS training program, all 132 participants completed the baseline assessments, which included the T-TPQ, HSPSC, and demographic questionnaire. Additionally, immediately after the program and two months later, all 132 participants completed the T-TPQ and HSPSC again, resulting in a retention rate of 100% (Table 2).

**Table 2:** Personal characteristics of the participants

Characteristics	n	%
Gender		
Male	37	28.0
Female	95	72.0
Marital status		
Single	35	26.5
Married	97	73.5
Education		
Bachelor’s degree	87	65.9
Technical degree	45	34.1
	Mean ± SD	
Age	21.92 ± 0.89	
Experience (months)	10.98 ± 6.41	

The mean score of teamwork perceptions increased from T0 (91.02 ± 31.10) to T1 (145.27 ± 12.87) and T2 (142.30 ± 15.74), with highly significant values in repeated measures two-way ANOVA (f = 307.06, *p* < 0.001) and paired t-tests results (p1 < 0.001, and p2 < 0.001). It also noted the large effect size of the TeamSTEPPS training program (η2p ≥ 0.14) (Table 3).

**Table 3:** Teamwork perceptions among participants throughout the different phases of the training program

Teamwork perceptions	Time points	Mean ± SD	f/p	P1	P2	η^2^p	Cronbach's α coefficient
A. Team structure	T0	19.17 ± 6.41	235.05	< 0.001	< 0.001	0.70	0.86
	T1	28.99 ± 2.65	< 0.001				
	T2	28.82 ± 3.09					
B. Leadership	T0	16.92 ± 7.09	216.61	< 0.001	< 0.001	0.62	0.90
	T1	29.13 ± 4.19	< 0.001				
	T2	28.11 ± 5.06					
C. Situational monitoring	T0	16.24 ± 7.62	322.31	< 0.001	< 0.001	0.71	0.94
	T1	29.54 ± 2.69	< 0.001				
	T2	28.89 ± 3.24					
D. Mutual support	T0	17.02 ± 7.13	297.68	< 0.001	< 0.001	0.69	0.88
	T1	28.60 ± 2.03	< 0.001				
	T2	28.22 ± 2.80					
E. Communication	T0	21.35 ± 3.85	236.76	< 0.001	< 0.001	0.64	0.87
	T1	29.02 ± 2.80	< 0.001				
	T2	28.27 ± 3.47					
Overall	T0	91.02 ± 31.10	307.06	< 0.001	< 0.001	0.70	0.92
	T1	145.27 ± 12.87	< 0.001				
	T2	142.30 ± 15.74					

p1: paired t-test between baseline and immediate after program */* p2: paired t-test between baseline and 2 months follow up

The mean of total patient safety culture score increased from T0 (98.42 ± 38.00) to T1 (150.50 ± 10.68) and T2 (151.11 ± 14.49), with highly significant *p* values < 0.001 for repeated measures two-way ANOVA and paired t-tests results (p1 < 0.001, and p2 < 0.001). It also noted the large effect size of the TeamSTEPPS training program on total patient safety culture (η2p ≥ 0.14) (Table 4).

**Table 4:** Patient safety culture among the participants throughout the different phases of the training program

Patient safety culture	Time points	Mean ± SD	f/p	P1	P2	η^2^p	Cronbach's α coefficient
1. Teamwork within unit	T0	10.04 ± 4.44	242.89	< 0.001	< 0.001	0.65	0.90
	T1	16.52 ± 1.21	< 0.001				
	T2	16.55 ± 1.68					
2. Supervisor/Manager expectations & actions promoting patient safety	T0	9.30 ± 4.14	264.13/	< 0.001	< 0.001	0.67	0.84
	T1	16.46 ± 1.86	< 0.001				
	T2	15.78 ± 2.12					
3. Organizational learning	T0	7.55 ± 3.02	230.46	< 0.001	< 0.001	0.64	0.78
	T1	12.32 ± 1.17	< 0.001				
	T2	12.23 ± 1.52					
4. Management support for patient safety	T0	8.44 ± 1.25	18.19	< 0.01	< 0.01	0.04	0.81
	T1	9.18 ± 2.06	< 0.01				
	T2	8.80 ± 1.66					
5. Overall perceptions of patient safety	T0	9.75 ± 3.69	90.59	< 0.001	< 0.001	0.41	0.79
	T1	13.80 ± 2.23	< 0.001				
	T2	14.36 ± 2.82					
6. Communication and feedback about error	T0	6.62 ± 3.73	115.00	< 0.001	< 0.001	0.47	0.81
	T1	10.98 ± 1.83	< 0.001				
	T2	11.17 ± 2.15					
7. Communication openness	T0	5.97 ± 2.94	96.40	< 0.001	< 0.001	0.42	0.92
	T1	9.54 ± 1.85	< 0.001				
	T2	9.44 ± 2.51					
8. Frequency of events reported	T0	7.00 ± 2.85	166.25	< 0.001	< 0.001	0.56	0.74
	T1	11.95 ± 2.09	< 0.001				
	T2	11.6 ± 2.46					
9. Teamwork across units	T0	9.48 ± 3.95	267.79	< 0.001	< 0.001	0.67	0.78
	T1	16.68 ± 2.17	< 0.001				
	T2	16.20 ± 2.62					
10. Staffing	T0	8.61 ± 4.19	44.17	< 0.001	< 0.001	0.25	0.75
	T1	11.13 ± 2.00	< 0.001				
	T2	12.39 ± 2.56					
11. Handoffs & transitions	T0	9.52 ± 4.04	241.84	< 0.001	< 0.001	0.65	0.89
	T1	16.01 ± 1.69	< 0.001				
	T2	15.90 ± 2.15					
12. Non-punitive response to errors	T0	5.93 ± 1.80	3.97	< 0.01	0.08	0.03	0.81
	T1	6.70 ± 2.40	< 0.05				
	T2	6.14 ± 2.14					
Total patient safety culture	T0	98.42 ± 38.00	226.28	< 0.001	< 0.001	0.61	0.93
	T1	150.50 ± 10.68	< 0.001				
	T2	151.11 ± 14.49					

p1: paired t-test between baseline and immediate after program / p2: paired t-test between baseline and 2 months follow up

## Discussion

The aim of this study was to evaluate the effect of TeamSTEPPS on the perceptions of teamwork and the culture of patient safety among recently graduated nurses.

The study revealed a significant increase in mean scores of TeamSTEPPS perceptions across multiple assessments. This discovery highlights the positive influence of the TeamSTEPPS training program in enhancing the perceptions of teamwork among these novice nurses, supporting hypothesis 1. The intervention's comprehensive effectiveness is evident across various domains, including team structure, leadership, situational monitoring, mutual support, and communication, indicating its ability to foster essential elements of effective teamwork within the nursing staff. These findings can be attributed to the controlled and targeted nature of the TeamSTEPPS program. TeamSTEPPS prioritizes evidence-based teamwork techniques, tools, and concepts tailored specifically for healthcare settings. Measures such as improving communication techniques, defining team responsibilities, and promoting mutual support directly address crucial aspects of successful teamwork [33].

The study highlights the importance of efficient teamwork in healthcare institutions for patient safety, care quality, and clinical outcomes. The positive impact of TeamSTEPPS on nursing graduates suggests its potential to foster a safety-oriented culture, leading to decreased medical errors, improved patient outcomes, and overall healthcare quality [2, 34].

The current study’s findings are consistent with prior research. For instance, Mohsen et al. [35] demonstrated a significant improvement in healthcare providers’ teamwork perceptions, encompassing various facets, alongside increased patient satisfaction upon implementing the TeamSTEPPS program in primary care units in Menoufia Governorate, Egypt. The study of Matzke et al. [3] reported improved teamwork and communication perceptions among registered nurses and patient care technicians in an academic Level I Emergency and Trauma Center located in the mid-Atlantic region of Virginia due to the TeamSTEPPS program. Furthermore, the pre- and post-study conducted by Dimarino demonstrated improved perceptions of teamwork and communication among the interdisciplinary healthcare team at the surgery center following the completion of customized TeamSTEPPS training [17]. Laura Dodge et al. highlighted improvements in staff perceptions of teamwork in ambulatory reproductive health care following TeamSTEPPS implementation [36]. Similarly, Obrnrader et al. observed perceived enhancements in teamwork and communication among emergency department staff due to TeamSTEPPS training [16].

However, contrasting findings were represented by Kwon and Duzyj who reported no substantial changes in perceptions of team dynamics or behaviors to promote patient safety among the physician and nurses at the regional perinatal center after teamwork training [37]. Similarly, the study conducted by Ahsan et al. found no noteworthy differences in attitudes and perceptions toward team communication between intervention and control groups [38]. Discrepancies in these results might be linked to differing sample sizes. Notably, Kwon and Duzyj collected the data from 20 physicians and 15 nurses before training and from 9 physicians and 20 nurses 6 months post- training [37]. However, the study by Ahsan et al. involved 28 nurses in control and intervention groups [38]. The dissimilarities in sample sizes might influence the sensitivity of detecting changes in perceptions, potentially explaining the divergent findings between these studies.

The study’s findings also indicated a significant increase in mean scores of patient safety culture across assessments, supporting hypothesis 2. These findings signify a positive influence of the Team STEPPS training program on the participants’ patient safety culture, with improvements observed across all dimensions. Multiple credible reasons account for the notable enhancements identified in the patient safety culture over the evaluation periods. Firstly, the TeamSTEPPS program equipped participants with practical resources and techniques to strengthen the implementation of patient safety measures, hence contributing to the observed enhancements across various aspects of patient safety culture. Secondly, the effectiveness of TeamSTEPPS implementation relies heavily on leadership endorsement and support. According to Montminy, robust leadership dedication can cultivate an organizational culture that places a high priority on patient safety and the importance of collaborative teamwork [39]. Therefore, the provision of support from leadership may have played a role in facilitating the successful integration of TeamSTEPPS concepts into routine healthcare practices, hence exerting a beneficial influence on the patient safety culture. The study’s results align with previous study by Mohsen et al. reported that the implementation of the Team STEPPS program resulted in significant improvement in healthcare providers’ patient safety culture [35]. Also, the study of Bonds reported that the implementation of the SBAR tool resulted in improvements in communication, teamwork, and the perception of patient safety culture among participants affiliated with surgical intensive care unit and anesthesia departments in a military teaching hospital [40]. A prior systematic review study found that treatments focused on teamwork and communication training enhance the safety culture in emergency department settings and might have a favorable impact on patient outcomes. Implementing safety culture programs can potentially decrease the occurrence of medical errors and adverse events [34].

The study found that TeamSTEPPS training significantly impacted teamwork perceptions and patient safety culture for two months after completion. This effect can be understood through Albert Bandura's social cognitive theory, which emphasizes the influence of social learning, observation, and cognitive elements on human behavior development. Initial observation and learning during training sessions can drive continuous reinforcing and practicing of learned behaviors. A supportive organizational atmosphere promoting learned strategies can foster these behaviors, aligning with social cognitive theory principles [22].

The study’s findings, supported by a previous study by Dodge et al. found that patient and staff views of teamwork significantly improved two years after the implementation of TeamSTEPPS in ambulatory reproductive health care centers [41]. This positive long-term effect was supported by one-group pre-post research by Aaberg et al. which also showed improvement in situational monitoring, mutual support, and communication dimensions of teamwork perceptions, two dimensions of patient safety culture (manager expectations and actions promoting patient safety, and communication openness) were improved after 12 months of completion in a urology and gastrointestinal surgery ward in Norway [18]. These findings are in line with those of Staines et al., who found that the teamwork concept and patient safety culture significantly improved for three of twelve dimensions in the intervention group, with a significant improvement remaining in one dimension when controlling for differences in baseline scores between implementation and control wards [20]. However, it is important to acknowledge certain limitations of this study. Firstly, the study was conducted exclusively at a single healthcare facility, limiting the generalizability of the findings to other settings. Additionally, the participants consisted solely of recently graduated nurses, which may not reflect the effects of the TeamSTEPPS training program on nurses with varying levels of experience. Future research should aim to include more diverse samples and encompass multiple healthcare facilities to validate and extend the results. Furthermore, the relatively short two-month follow-up period poses a challenge in assessing the long-term retention of the TeamSTEPPS training material by the participants. Conducting longitudinal studies with extended follow-up periods is necessary to gain a comprehensive understanding of the long-term effects. Moreover, it is important to note that this study utilized a one-group pre- and post-test design, providing preliminary evidence of the benefits of TeamSTEPPS. To enhance the robustness of future evaluations, it is recommended to incorporate a control group, enabling more rigorous and conclusive results.

## Conclusions

In summary, this study employed a quasi-experimental pretest–posttest design with a single group. The convenience sample consisted of newly recruited nurses from a university hospital. The main objective of the study was to examine the effectiveness of the TeamSTEPPS program in improving newly graduated nurses' perceptions of teamwork and patient safety culture. The study findings contribute novel evidence that implementation of a TeamSTEPPS training program can significantly improve perceptions of teamwork and patient safety culture among newly graduated nurses over time. Specifically, enhancements were observed across total scores on validated assessments as well as within individual domains measuring core outcomes up to 2 months post-intervention. These findings highlight the effectiveness and sustainability of the TeamSTEPPS program in promoting positive changes in teamwork perceptions and patient safety culture among newly graduated nurses.

## Implications of the study

Nurse managers should introduce TeamSTEPPS programs for new nurses, helping them adapt to the culture of patient safety for better outcomes. They should create conducive work conditions that support the long-term integration of teamwork skills. This involves ensuring program fidelity, booster training, and supportive organizational processes that reinforce learned strategies in clinical practice. Furthermore, incorporating TeamSTEPPS into undergraduate nursing education can enhance readiness, build prelicensure training, and cultivate comprehensive skills for real-world application. Qualitative research can offer valuable insights by exploring participants’ perspectives and how their skills are integrated into everyday work.

## Data Availability

The quantitative datasets used and analyzed in this study are available from the corresponding author on reasonable request.

## Abbreviations

AHRQ: Agency for Healthcare Research and Quality
HSPSC: Hospital Survey on Patient Safety Culture
TeamSTEPPS: Team Strategies and Tools to Enhance Performance and Patient Safety
T-TPQ: Team STEPPS Teamwork Perceptions Questionnaire
